# Applications of Mass Spectrometry in Polymer Analysis: Use of GC-GC-High Resolution MS to Identify Photo- and Oxidative Degradation Products of BPA-Polycarbonate

**DOI:** 10.6028/jres.093.090

**Published:** 1988-06-01

**Authors:** Woodfin V. Ligon, Arnold Factor, Ralph J. May

**Affiliations:** General Electric Company, Corporate Research and Development, Schenectady, NY 12301

## Introduction

A full understanding of any chemical process usually requires the complete elucidation of all of the reactants involved and all of the products produced. Even products produced in very minor quantities can be important because they may provide mechanistic clues.

The photooxidative degradation of polymers is an economically important process. If we are designing windows of a clear plastic then stability to light is all important in order to extend useful life. If we are designing certain kinds of single-use containers then we may wish them to degrade rapidly in order to minimize the potential for environmental contamination.

Identifying the products of the photooxidation process is, however, an unusual challenge for the analytical chemist. Especially in the early stages of the process, the products are likely to remain bound to the polymer backbone whose molecular weight may typically lie in the tens of thousands. This means that we are really talking about a very small change in a very large molecule. Accordingly these products are often not amenable to the usual battery of organic analytical methods such as nmr and mass spectrometry. Mass spectrometry is limited by the high molecular weight and nmr (and often ir) are limited by the low effective “concentration” of the altered functional groups. Moreover, these processes are typically exceedingly complex—commonly producing 30 or 40 products. Relatively global surface techniques such as electron spectroscopy for chemical analysis (ESCA) while providing some insight into mechanism cannot possibly provide the detailed speciation needed to fully understand mixtures of this complexity.

A final impediment involves the fact that photooxidation is a surface phenomenon and, for this reason, altered products may have to be isolated from layers only few micrometers thick. As a result the total mass of “sample” available to the analyst may be quite small.

In this paper we discuss our approaches to understanding the photooxidation of Bisphenol-A (BPA) polycarbonate. A detailed experimental procedure will not be presented here and is in press elsewhere [1]. In keeping with the theme of this conference, this paper will concern itself with the relatively philosophical and strategic questions of the design of the analytical method for these trace components.

## Discussion

The carbonate linkage of BPA-polycarbonate provides an attractive strategy for recovery of the photooxidatively altered portions of individual macromolecules. Simple base hydrolysis of the carbonate produces largely BPA and carbonate salts together with trace quantities of the various photooxidative products [2]. However, there is some concern that not all of the degradation products survive treatment with strong base. As an alternative, it was felt that degradation with lithium aluminum hydride (LAH) would both destroy the carbonate linkage (producing methanol) and reduce materials in a highly oxidized state (quinones, acids, etc.) to less polar, more easily characterized and more stable forms. However, it was recognized that use of a reduction step introduces some ambiguity in terms of speciation of the original photooxidation products. In order to facilitate speciation, it was decided to reduce aliquots of the altered polymer with both LAH and lithium aluminum deuteride (LAD). It was reasoned that if mass spectra of the LAH and LAD product mixtures were compared, then one could conclude that materials differing by one amu were derived from ketones or aldehydes whereas materials differing by two amu were derived from acids or esters. Reduction products of quinones would not retain the hydride and therefore would not retain the label.

Having decided on a degradation strategy, about 30 mg samples consisting of 10 *μ*m thick slices of altered polymer were obtained from the surface of a photo-aged outdoor exposed specimen. In addition samples were taken from below the surface to act as controls on the analytical procedure. These samples were all subjected to LAH and LAD reduction.

Hydride reduction provided the photooxidation products in a form which was amenable to traditional techniques such as GC-MS. However we were then faced with the presence of a relatively large quantity of BPA that acted as a diluent for the products of interest which were present in trace quantities. High resolution gas chromatography could not be used directly because the amount of the major component (BPA) which could be injected without degrading chromatographic performance was limited to several micrograms. When this quantity of BPA was injected, the minor components were present in such small amounts that high resolution mass spectrometry was not practical. Although liquid chromatography could have been utilized to separate the BPA, it was reasoned that a two-stage gas chromatographic (GC) separation would provide many more theoretical plates and therefore minimize the probability that photo-products which very closely resembled BPA would be lost by co-elution. Furthermore a two-stage GC separation is much faster and highly efficient.

Using two-dimensional GC-MS methods, which have been described elsewhere [3], we obtained a primary separation by injecting milligram size aliquots of the product mixture onto a packed primary GC column. The entire effluent of this column was collected in a cold trap with the exception of the solvent and the PBA peak which were both vented. The components collected in the cold trap were then rechromatographed on a high resolution fused silica capillary and in separate analyses both low resolution and high resolution mass spectrometry data were obtained. Using this technique about 40 photooxidation products were resolved and characterized.

These data allowed the identification of products arising from ring oxidation, ring attack, side chain oxidation, and photo-Fries reactions.
